# Supplementary material for: Parental Perceptions and Exposure to Advertising of Toddler Milk: A Pilot Study with Latino Parents
Source: Int J Environ Res Public Health. 2021 Jan 10;18(2):528. doi: 10.3390/ijerph18020528 (PMC7827454; doi:10.3390/ijerph18020528)
Supplement: Supplementary file 1 [file ijerph-18-00528-s001.pdf]

Table S1. Toddler milk survey items

Prompt	The next questions are about toddler milks. Toddler milks are intended for children at least 12 months old. They are different from formulas like Enfamil and Similac that are for babies younger than 12 months old. The picture below shows two examples of toddler milks.<br><br>[Images of Nido Kinder 1+ and Enfagrow Toddler Next Step were shown]	
Familiarity	Before being in this study have you ever seen toddler milks in grocery stores, convenience stores, or stores like WalMart?	1=Yes<br>0=No [skip to reasons]
Ever purchased	How many times before being in this study have you bought toddler milks for your children?	0=Never [skip to reasons]<br>1=1-2 times<br>2=3-4 times<br>3=5-9 times<br>4=10 or more times
Conversations	The next questions are about talking with people about toddler milks.	
Conversations **Adapted from (Hall et al., 2015)**	Who have you talked to about toddler milks? (Check all that apply)	1=My spouse or significant other<br>2=My child or children<br>3=Other family member<br>4=Friend<br>5=Co-worker<br>6=Health care provider<br>7=Someone I did not previously know<br>8=Other<br>9=I did not talk to anyone about toddler milks [skip to reasons]
Conversations **Adapted from (Hall et al., 2015)**	Think about the conversations you have had about toddler milks. What came up during these conversations?	[open-ended]
Reasons	[Images of Nido Kinder 1+ and Enfagrow Toddler Next Step were shown again]<br><br>Why do you think parents would want their children to drink toddler milk? (Check all that apply)	1=To save money<br>2=To help children's growth<br>3=To help children's brain development<br>4=To provide nutrients and vitamins<br>5=To help picky eaters<br>6=Because toddler milk is healthy<br>7=Because children like the taste of toddler milk<br>8=Because children do not like the taste of regular milk

		9=Because toddler milk is healthier than regular milk<br>10=Because parents grew up drinking this kind of milk<br>11=Other: _______
Reasons	Why else would parents want their children to drink toddler milks?	[open-ended]
Exposure to advertisements	In the past 30 days, have you seen or heard any advertisements for toddler milks?	1=Yes<br>0=No [Skip to Familiarity with Nido]
Exposure to advertisements	Which brands of toddler milks have you seen advertised in the past 30 days? Please be as specific as possible.	[open-ended]
Exposure to advertisements	In the last 30 days, where have you seen or heard advertisements for toddler milks? (Check all that apply)	1=Spanish-language Television<br>2=English-language Television<br>3=Coupons<br>4=Magazine or newsletter<br>5=Convenience store or gas station<br>6=Supermarket or grocery store<br>7=Social media (such as Facebook, Instagram, or Youtube)<br>8=Mom or parenting website or blog (NOT social media)<br>9=Retailer websites (WalMart, Amazon, Target)<br>10=Other: ______
Prompt	The next questions will ask about the beverage in this picture.<br><br>[Image of Nido Kinder 1+ was shown]	
Familiarity with Nido	Have you ever seen this product before being in this study?	0=No [Skip to perception of ingredients]<br>1=Yes
Ever purchased Nido	Have you ever purchased this product before being in this study?	0=No<br>1=Yes
Perception of ingredients	What ingredients do you think are in this beverage?	[open ended]
Perceptions of healthfulness<br><br>**Adapted from (Bollard et al., 2016)**	How healthy or unhealthy would it be for a toddler to drink this beverage every day?	1=Unhealthy<br>2<br>3<br>4<br>5=Healthy
Perceptions of healthfulness	Why did you rate this product as healthy or unhealthy?	[open-ended]

Perceived amount of added sugar	The next questions will ask you about added sugar. By "added sugar," we mean any sweetener that adds calories, such as sugar, corn syrup, cane sugar, high fructose corn syrup, or honey.	
Misperception that drink does not contain added sugar	Do you think this beverage has added sugar?	1=Yes<br>0=No [coded as misperception]
Prompt	We have a few more questions about this beverage.<br><br>[Image of Nido Kinder 1+ was shown again]	
Claims	This product says "Helps support healthy growth." What does the phrase "Helps support healthy growth" tell you about the product?	[open-ended]
Claims	This product says "Immunity." What does the word "Immunity" tell you about the product?	[open-ended]
